# Solution-focused approaches in adult mental health research: A conceptual literature review and narrative synthesis

**DOI:** 10.3389/fpsyt.2023.1068006

**Published:** 2023-03-30

**Authors:** Lauren Jerome, Philip McNamee, Nadia Abdel-Halim, Kathryn Elliot, Jonathan Woods

**Affiliations:** ^1^Unit for Social and Community Psychiatry, Wolfson Institute of Population Health, Queen Mary University of London, London, United Kingdom; ^2^Unit for Social and Community Psychiatry, Newham Centre for Mental Health, East London NHS Foundation Trust, London, United Kingdom; ^3^Department of Clinical Psychology and Psychological Therapies, The University of East Anglia (UEA), Norwich, United Kingdom

**Keywords:** solution-focused, adult mental health, conceptual review, narrative synthesis, therapeutic approach

## Abstract

Solution-focused approaches are one approach to treatment used in a wide variety of settings in modern mental healthcare services. As yet, there has been no overall synthesis of how this approach is understood in the adult mental health literature. This conceptual review aimed to synthesize the ways that solution-focused approaches have been conceptualized and understood, within the adult mental health literature, in the five decades since their conception. A systematic search followed by multiple techniques from the narrative synthesis approach were used to develop a conceptual framework of the extracted data. Fifty-six papers published between 1993 and 2019 were included in the review. These papers spanned a variety of clinical contexts and countries, but despite this the underlying key principles and concepts of solution-focused approaches were remarkably similar over time and setting. Thematic analysis of extracted data outlined five key themes relevant to the conceptualization of this approach. This conceptual framework will help support clinicians using solution-focused techniques or therapies by giving them a coherent understanding of such approaches, by what mechanisms they work, and how key principles of this approach can be utilized in adult mental health settings.

## 1. Introduction

One in six adults in England were found to have a common mental health disorder in a 2014 National Statistics report, and one third of them reported current use of a mental health treatment, with medication being the most common form of treatment reported (1). However, health care services including GPs, inpatient, outpatient, and community services were all utilized for mental health treatment where a range of psychotherapeutic approaches were also being delivered (1). Solution-focused therapy is one such approach that has been gaining increasing popularity worldwide since its conception. Specifically in the UK, where BRIEF became the first team to practice Solution-Focused Brief Therapy (SFBT) in the 1980’s (2). Solution-focused therapies are now being practiced in a wide variety of settings (3), including being recommended in the Nursing and Midwifery Council proficiency standards for newly qualified nurses (4) and in psychiatric practice (5).

The premise of a solution-focused approach was first described in 1974 by Watzlawick, Weakland, and Fisch in their Brief Therapy approach (6), where they described their belief that effective change can be brought about by enacting change in the present, without a need to necessarily understand “why” or the cause of a problem. They suggested the focus of therapy should be on investigation of previously attempted solutions to a problem to identify what is maintaining it, constructing clear concrete goals to define the change to be achieved, and finally formulating and implementing a plan to achieve such change. This approach was further developed in the 1980s, and most famously in de Shazer’s development of SFBT (7). SFBT builds on Watzlawick, Weakland and Fisch’s (6) approach, being oriented toward building solutions by identifying the client’s resources, and when something is found that works, the client should do more of it (8). It’s generic approach means that it is suitable for most therapeutic settings (5). De Shazer and Berg (7) outlined four characteristic features of SFBT, which they said should be present to ensure SFBT is taking place; (1) asking of the miracle question (a question that asks the client to describe how life might be different for them if something miraculous happened), (2) at one point in the session the client should be asked to scale something from 0–10, (3) the therapist should take a break, and (4) after returning from the break the therapist should provide a compliment and suggest a homework task. However, it is unclear how closely solution-focused approaches being practised today follow this original conceptualization, and whether these four features are still considered essential.

A review of SFBT controlled outcome studies by Gingerich and Peterson (3) found strong evidence for the effectiveness of SFBT across a large number of settings and populations, supporting de Shazer and Berg’s claims of widespread success. As a result of this review, the authors suggested there are six techniques core to the SFBT method: including specific goals, the miracle question, scaling questions, searching for exceptions, compliments, and homework. Of the 43 studies included in their review, they found 33 implemented all of these six techniques, and only three implemented three or fewer, concluding treatment fidelity is high. This demonstrates that since de Shazer and Berg outlined their characteristic features of SFBT, there has been some shift in what is considered to be integral to this approach.

While Gingerich and Peterson’s review provides recent evidence for the effectiveness of the techniques implemented within SFBT, SFBT is just one form of therapy within the wider solution-focused approach. There remains a need for a synthesis of the conceptualizations of the overall solution-focused approach in adult mental health research. Such conceptual work would allow common attributes of the approach to be identified by exploring the various ways in which this approach has historically been described. It would also uncover how the term “solution-focused” has been used, changed, and amended since its original conception and description. Moreover, building a conceptual framework of solution-focused approaches would provide clinicians and researchers with the theoretical underpinnings to enable a deeper understanding of the mechanism by which interventions based on a solution-focused approach function, and how key principles of the approach can be utilized.

Therefore, the aims of the current review are to undertake a conceptual review and narrative synthesis of solution-focused approaches in adult mental health research and to synthesize published concepts of solution-focused approaches into a systematic conceptual framework. The questions we aim to answer are:

(1) what are the common attributes defining interventions conceptualized as “solution-focused”? and, (2) how has the phrase “solution-focused” been used historically in adult mental health literature?

## 2. Materials and methods

For this review, we were not aiming to necessarily include every publication that cited a solution-focused approach, instead we were interested in including a breadth of papers from across the research literature to investigate widely whether the term “solution-focused” is applied similarly or understood differently by researchers representing distinct research areas and specialisms. As such, a conceptual review with narrative synthesis was deemed the most appropriate approach, with a systematic search to capture as many different publications as possible. We followed recommendations made by Lilford et al (9) which state rather than exhaustively searching for every publication, a wide and disparate source of evidence should be sought. We included several steps in our search process, described below, to ensure it was systematic, robust, and reflected an extensive range of the available literature.

The main review team consisted of three research assistants (LJ, NAH, KE), one senior researcher (PM), and one trainee clinical psychologist (JW). All authors had some exposure to training in solution-focused therapy and/or conducting research into interventions based on the principles of solution-focused therapy. The approach to this review and its emerging findings were discussed on several occasions with a wider group of roughly 30 researchers and clinicians, some of whom have clinical and/or research expertise in solution-focused approaches, and who have experience in conducting conceptual reviews.

### 2.1. Protocol and registration

In accordance with PRISMA guidelines, the methods of this paper were pre-specified in a registered protocol (PROSPERO: CRD42018090195).^1^

### 2.2. Eligibility criteria

Full text papers, published in peer-reviewed scientific journals, were included if they included a detailed outline of a self-described “solution-focused” approach. “Self-described” here means either the name of the approach referred to “solution-focused” or “solution-focused” was cited as a descriptor or label. This was in order to better understand how “solution-focused” as a term and an approach was described in the literature, and to analyze what the key conceptual components of these approaches were. As such, the paper had to include sufficient detail to allow a succinct description of the approach to be extracted. Furthermore, the approach had to be delivered/described in the broad context of adult mental health care, including but not limited to primary care, inpatient, and/or community care. Primary studies were included if there were at least three participants with a self-described mental health issue, as were secondary research studies synthesizing existing literature. Only papers available in the English language were included. Any research involving a sample of mixed ages were included if over 50% of the sample were aged over 18.

Additionally, we included textbooks (where they were available) or guidelines, written by professionals working within a mental health context, describing the model, including original descriptions and characteristics of the model, when they were either recommended by key experts in the field or identified as commonly occurring in the reference lists of articles recommended by key experts.

Texts were excluded if they did not present a clear conceptualization of the solution-focused approach. Papers reporting on primary research with less than three participants were also excluded as the focus was on looking at general use and delivery of the approach, this exclusion criteria also applied to case studies and studies reporting first-person experiences or those using auto-ethnography. Further, papers reporting on solution-focused approaches applied to a non-mental health population were also excluded, as was grey literature, such as PhD theses and conference papers.

### 2.3. Data collection

First, key experts in the field were contacted to identify seminal publications. This was accompanied by a search of Google Scholar using broad search terms to identify relevant publications to act as marker papers in the subsequent systematic search.

Next, three electronic bibliographic databases, PsycINFO, EMBASE, and Web of Science, were searched from 1974, the year the first text on a solution-focused approach was published (6), to present. PubMed was also initially included however no additional unique publications were identified.

The following keywords were searched for terms characterizing:

- solution-focused approaches ((“solution-focus*” or “solution-orient*” or “solution-driven” or “solution-focus* brief”)) adj5 (therap* or psychotherap* or approach* or intervention*)) AND.
- mental health (mental disorders/or ((mental* or psych*) adj2 (health* or disorder* or disease* or deficien* or ill* or problem* or condition* or treat*).

After removing duplicates, titles and abstracts were screened for potentially relevant papers. The full texts of the remaining papers were then screened, and inclusion was based on the eligibility criteria described in section “Eligibility criteria.”

Finally, reference list screening and forward citation tracking was conducted for each significant paper identified by key experts, and each eligible publication to be included in the review, to identify additional relevant papers.

### 2.4. Data analysis

**Figure 1:**
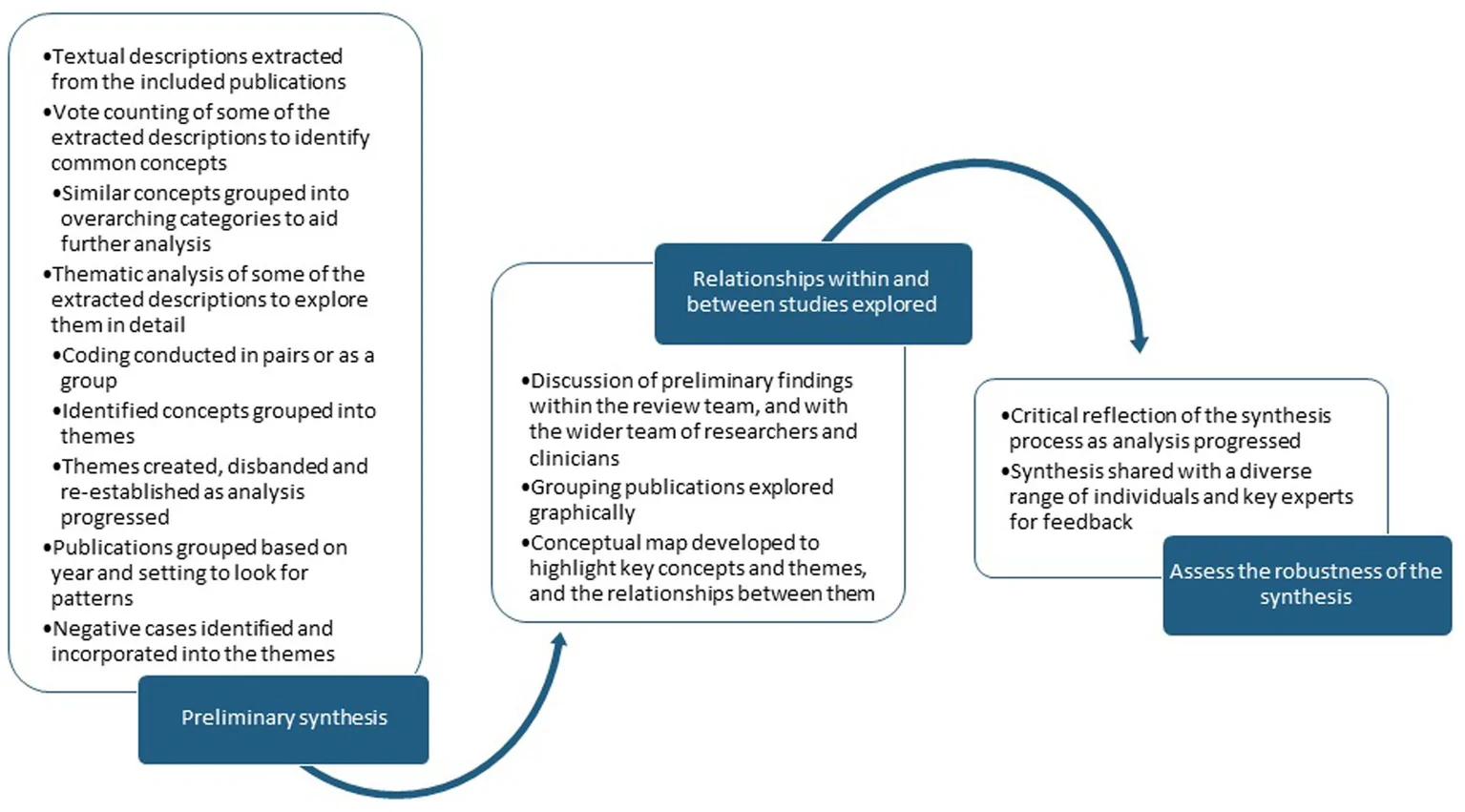
Schematic providing an overview of the narrative synthesis process, and the specific techniques we utilized in this review.

We conducted a three-stage narrative synthesis of the data following the guidance of Popay et al (10) to integrate the findings into a conceptual framework. As recommended by Lilford et al (9) these stages were part of an iterative process and had some overlap. Figure 1 provides an overview of the synthesis process.

First, a preliminary synthesis was developed using techniques described by Popay et al (9) to identify the main themes and subthemes in the publications.

(1) Initially, textual descriptions of a variety of information from the publications were extracted and then tabulated to aid further analysis.
(2) Vote counting was then performed to produce an initial description of patterns across the included publications, the findings of which were then further explored (9). Vote counting was performed on the following elements: the name of the approach; setting; theoretical background; and any key definitions and steps given. As there were a lot of unique concepts identified for theoretical background and key definitions/steps, any that could reasonably be grouped to aid further analysis were grouped under overarching categories; e.g., the miracle question, the tomorrow question, and asking the client to envisage their ideal situation, were all grouped under “focusing on the client’s future”. These categories were created deductively through discussions within the review team and our knowledge of the literature. All unique and distinct references to each category were classed as a vote, as such one paper may have multiple votes for the same category where they describe several different concepts that come under the same category.
(3) A thematic analysis was then conducted to explore, in detail, the authors’ definition of solution-focused, the categories identified through vote counting the key definitions and steps, and practitioner characteristics. For the authors’ definition of solution-focused and the practitioner characteristics, the review team met in person and discussed the textual descriptions extracted for each paper for those elements, and wrote any concepts that were identified on post-it notes. As we progressed through the papers, post-it notes were grouped into themes. To analyze the categories identified from vote counting of key definitions and steps, the full descriptions of all the unique concepts grouped under each category were uploaded, by category, into NVivo 12. Two researchers analyzed each category by creating codes within NVivo. After all categories had been coded, the review team met to discuss their findings and assimilate their ideas into themes and subthemes. These findings were discussed in relation to the themes identified from the analysis of the definition of solution-focused, as there was a lot of overlap in the concepts identified.
(4) In the final part of this preliminary synthesis, publications based on publication year and setting were grouped together in order to look for patterns within and across groups.

As analysis progressed, we began to develop our ideas of themes and subthemes present across the publications. Often we would return to concepts identified in analysis of a different element, or an earlier phase of analysis, and add further subthemes or depth to it, or disband themes entirely and re-establish new themes based on our ideas emerging from subsequent analyses. Many of our initial ideas merged into larger, overarching themes as analysis progressed and our ideas surrounding these concepts developed.

As well as synthesizing our findings into common themes and attributes, we were also interested in identifying negative cases, where a concept opposed those that were commonly identified. When we came across such cases, the review team discussed these and how they related to the themes we were developing. In the same way we would re-evaluate, disband and create new themes based on similar concepts identified, the themes were developed containing these opposing ideas.

Second, to explore relationships within and between studies, there was continuous discussion among the review team, other researchers and clinicians throughout the process. The initial findings that came from grouping publications were presented graphically to further explore the patterns found within and between the groups. Each member of the review team was involved in the preliminary synthesis, and in discussion of the identified themes, categories, and patterns within and between groups. The collaborative nature of our synthesis enriched our findings by ensuring we considered different views and interpretations, and contributed to the iterative nature of our analysis, as different insights developed our understanding of the concepts we identified. Finally, we created a conceptual map to highlight the key concepts and themes identified and the relationships between them. This final step brought together the results of our analysis and synthesized our ideas into one conceptual framework. This step also produced a visual representation of the concepts which further aided their definition, and established their relationships to one another.

Third, in order to assess the robustness of the synthesis we reflected critically on the synthesis process as we proceeded with our analysis. As we progressed through the analysis we considered how our knowledge of the literature, the analysis process we chose to use, and our individual preconceptions of solution-focused approaches may influence our identification of concepts and themes and how we synthesize them, and included this reflection in our discussions of the findings and our synthesis into the conceptual framework. We also shared our synthesis with a diverse range of researchers and clinicians, and with key experts, for feedback, to enable us to consider a range of views outside of the review teams.

We chose not to assess the quality of the included papers as we were interested in how approaches described as “solution-focused” are conceptualized in the literature, regardless of whether these approaches are then used in high quality research or not, we merely wanted to capture how the term is understood.

## 3. Results

### 3.1. Publication characteristics

**Figure 2:**
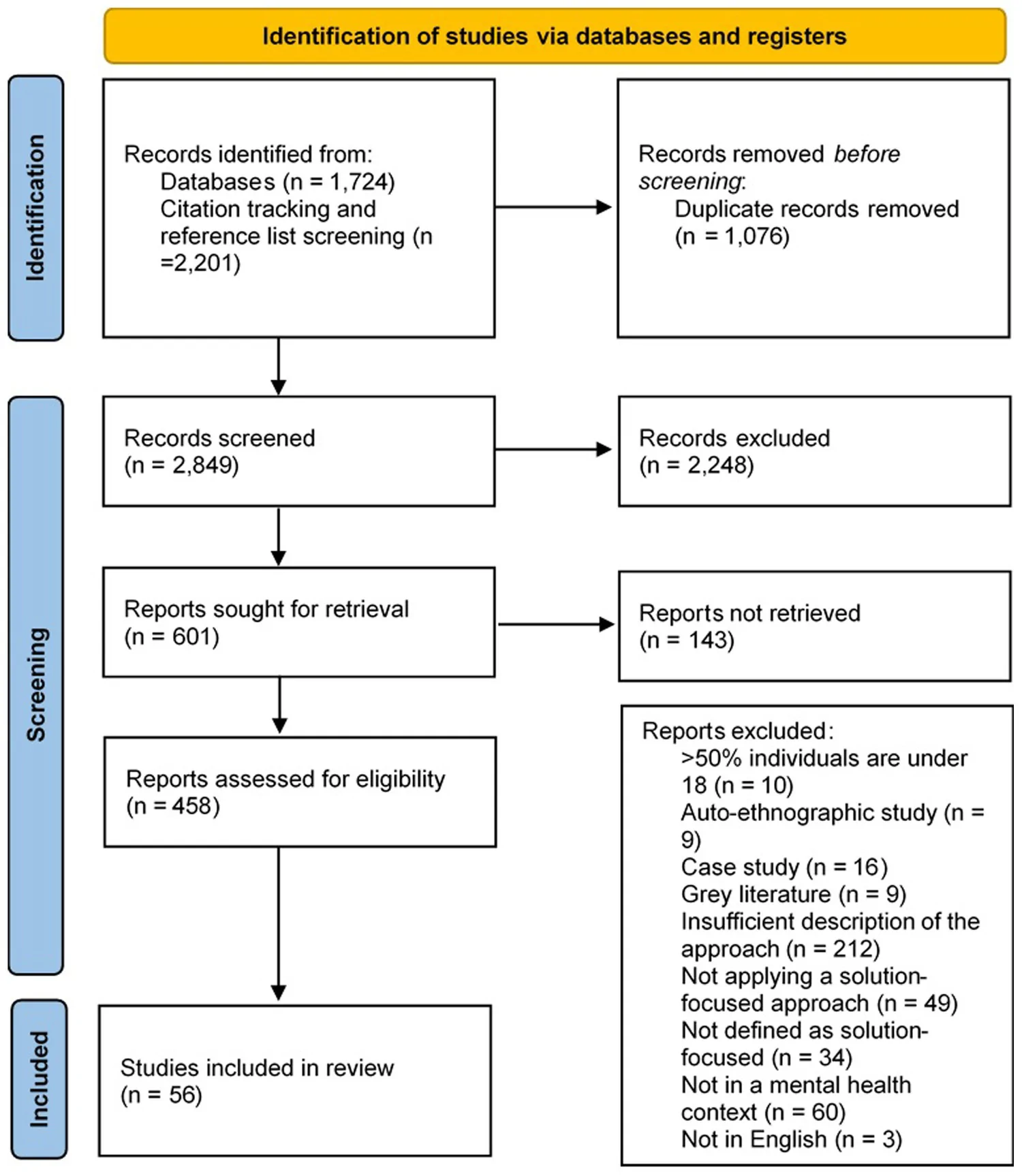
PRISMA 2020 flow diagram for new systematic reviews. From: Page et al. (11).

Figure 2 shows our search and selection process for the papers included in this review.

We included 56 papers published between 1993–2019. Thirty-five studies (65%) were therapeutic practice research papers, and twenty-one studies (35%) were interventional investigative research papers. Over half of the papers included in this review were published in the USA (52%), and the majority of the other included papers were published in Western countries. There were only two papers from non-western countries (Israel and Iran) included in the review (12, 13). Regarding the study setting for the investigative research papers, eight studies were conducted within community mental health teams (38%), four studies occurred in primary care settings (19%), and three studies within an inpatient setting (14%). The remaining papers represent one study conducted in each of; a nursing home facility (5%), a web based SFBT chat room (5%), a non-profit community group (5%), an addiction service (5%), a school counseling team (5%), and a healthcare organization (5%). These findings demonstrate the wide applicability of solution-focused approaches in a number of different settings and contexts. A full and detailed list of all included papers is provided in Supplementary material S1.

The papers included in this review describe clinicians from a variety of disciplines as delivering their solution-focused approach. For the purposes of this review for the remainder of our findings we will refer to the individual delivering the approach as the “practitioner,” which encompasses any individual delivering the approach, including but not limited to therapists, nurses, counselors etc., and the recipient of the approach as the “client.”

### 3.2. Descriptives

#### 3.2.1. Vote counting

Vote counting the name of the approach used in the papers demonstrated that all except two papers called their approach some form of “solution-focused therapy,” usually reflecting the specific context of the approach, e.g., solution-focused nursing. One paper referred to their approach as the “strengths perspective” (14), and another as “empowerment based practice” (15). Although our search terms limited inclusion of papers to using the term “solution-focused,” so this finding is not a surprise, the ubiquity of the term was widespread.

Vote counting identified a number of different theoretical backgrounds described as the basis for the solution-focused approach. However, the most common theoretical background given (*n* = 18) was self-described as a “solution-focused” theory. This was followed by post-modern theories (*n* = 13), Ericksonian hypnotherapy (*n* = 4) the strengths perspective (*n* = 4), hope theory (*n* = 2), and the systems perspective (*n* = 2). The remaining 14 theories identified were unique, i.e., not described in any other paper, and many papers described more than one theory as influencing their approach. In contrast, 14 papers did not name any theoretical background for their approach. A full list of the theoretical backgrounds identified is in Supplementary material S2.

**Figure 3:**
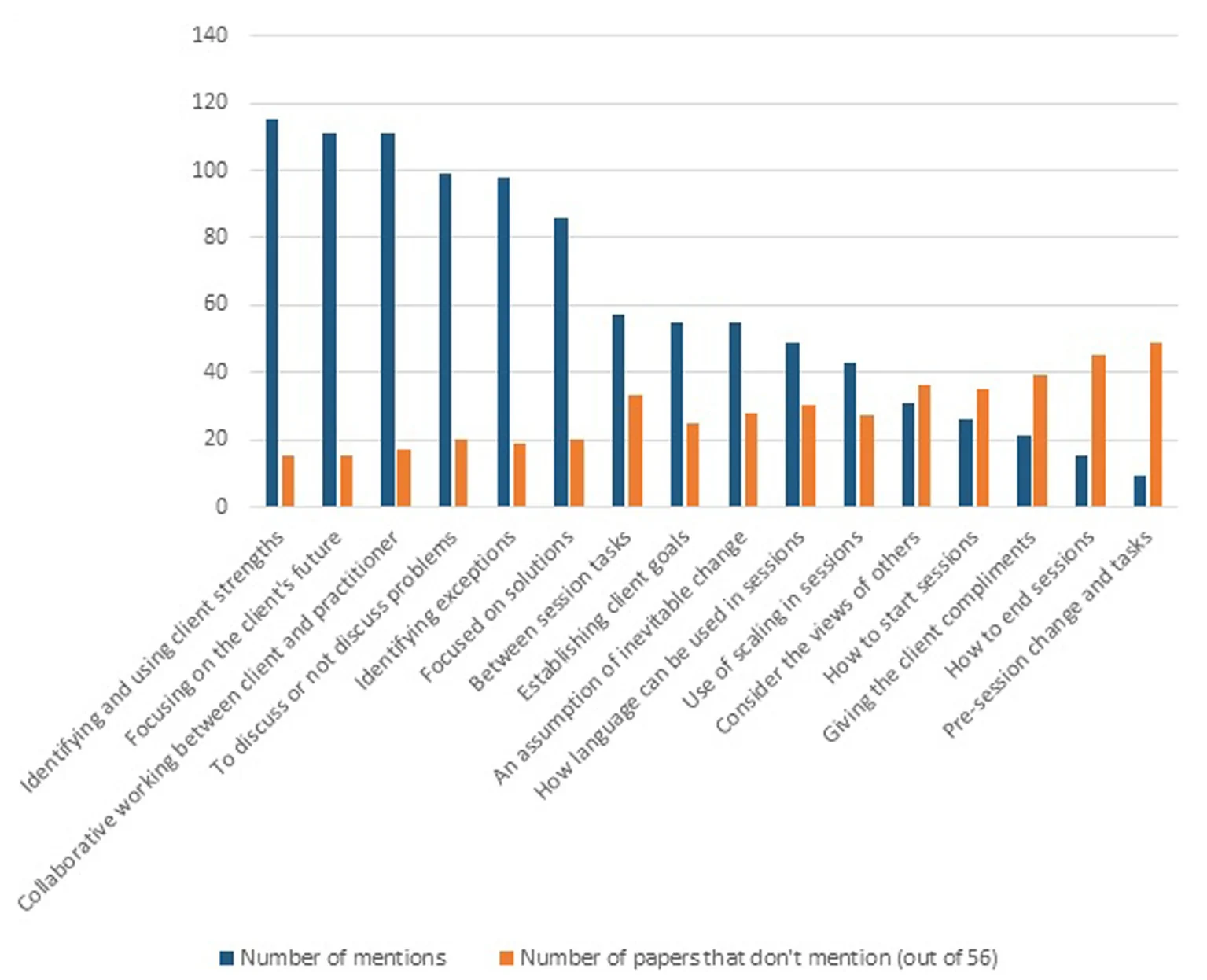
Graph showing key components identified from vote counting of the key definitions and steps described in the included papers. The number of times each component is mentioned is displayed, as well as the number of papers that do not mention each component at all.

Vote counting of the categories that derived from the key definitions and steps identified several key components that were consistently described in the included papers. Figure 3 shows the key components that we identified and how many times they were mentioned. Often multiple distinct concepts within a component were described within a paper, and so a paper could have more than one vote for a single component. Figure 3 also shows in contrast how many papers did not mention these components at all. Although some components were consistently mentioned more than others, none were mentioned in every paper. The identified components were explored further through thematic analysis, contributing to our resulting themes described in section “Themes.”

#### 3.2.2. Groupings

Grouping the included papers by their decade of publication identified some differences in how prevalent the key components, identified through vote counting, were presented in the included publications. The concepts of tasks, goals, scaling, a solution focus, and language were mentioned more often in more recent papers. In contrast, the concepts of compliments, exceptions, problems, pre-session work, beginning sessions, ending sessions, strengths, and collaborative working were mentioned less often in more recent papers. Compliments, for example, were mentioned an average of 1.1 times per included publication in those published between 1990 and 2000, compared to an average of 0.3 times in papers published from 2010 onwards. On the other hand, the concepts of a future focus and the views of others remained fairly consistent in their prevalence over time.

As we conducted the thematic analysis we were mindful of noticing any differences in how the themes we identified were presented over time, and whether any were particularly prevalent in one decade compared to another. In contrast to our findings from the categories identified from vote counting, described above, we did not identify any differences over time in our themes.

When comparing papers based on setting the concepts of beginning sessions, change, problems, a solution focus, strengths, collaborative working, scaling, exceptions and the views of others were mentioned more often in community settings than in other settings. In counseling settings, the views of others and scaling were not mentioned at all, and language was not mentioned in any papers in inpatient settings. However, there were very few papers in defined settings outside of community settings, and a number of papers did not clearly describe the setting their approach was conducted in, instead often just describing it as being conducted by a “therapist.” This limits the usefulness of grouping the papers by setting and these findings, as there were too few examples in the settings outside of community settings to make any confident conclusions.

### 3.3. Themes

In total, there were five themes that resulted from the thematic analysis of the descriptive elements of the extracted data. These themes encapsulated the key and distinctive aspects of solution-focused approaches that were described in the adult mental health literature. Some of the themes relate to the general philosophy of the approach (“Perceived as moving away from traditional approaches”; “problems vs. solutions”), one related to the specific techniques, tools and processes recommended (“Solution-Focused Tasks”), while others related to the role of key stakeholders in the process (“views of others”; “practitioner characteristics”). The themes will be outlined in full below with reference to the extracted data.

#### 3.3.1. Perceived as moving away from traditional approaches

The idea that the main focus of therapy should be directed toward solutions rather than on problems was seen as a radical idea in the 1970s when first introduced by Watzlawick, Weakland and Fisch (6). This idea was then developed further still by de Shazer & Berg in the 1980s. Although it may no longer be viewed as so radical in contemporary discussion, many of the included papers still positioned solution-focused approaches as being non-traditional. The unique core tenet of shifting away from problems and instead centralising solutions in order to be proactive in mental health promotion, rather than reactive, was still viewed as distinguishing it from other major approaches in psychotherapy.

“An effort to reverse traditional psychotherapy practice by shifting the focus of treatment from problems to solutions” (16, p. 455).

The shift from “traditional approaches” was not just limited to a focus on solutions, but also included the brevity and focus on achievable outcomes, as well as a shift from focusing on pathology to general mental health, particularly in regards to being less concerned with diagnostic criteria and specific symptoms.

“Traditionally, counselor and client are focused on pathology and dysfunction. From a solution-focused perspective, emphasis is placed on normalizing behaviors and ways of thinking as well as reframing the client’s situation and behaviors in ways that illuminate her or his strengths and resources” (17, p. 155).

This non-specificity meant such approaches could be flexibly applied across differing clinical and cultural contexts. As a result, solution-focused approaches were often conceptualized as “contemporary,” with their generic yet coherent principles being seen as culturally compatible.

“The model sits well with the contemporary practice of empowerment and partnership and is, by definition, sensitive to differences of culture” (18, p. 34).

Linked to this theme was the central principle within solution-focused approaches of “client knows best,” where the client is seen as an expert. This goes beyond traditional assumptions of expertise by experience and instead sees the client as an expert in all aspects of their lives while the practitioner adopts a “not knowing stance.”

“Clinicians do not assume they have an a priori expertise sufficient to objectively categorise and solve client problems. Clients are the only knowers and experts of their individual experiences, realities, and aspirations. Clients define the goals for treatment and thus are more likely to assume responsibility for working for a better and more satisfactory life” (19, p. 37).

Only the client has the ability to proactively identify the problem/s to work on, with no input or assumptions from the practitioner about what the problem may actually be. The way the client understands the problem is ultimately how the practitioner should understand it too, with caution against too much interpretation or analysis by the practitioner. The practitioner’s key responsibility is to keep the therapeutic process on track, by “*leading from one step behind*” (5, p. 298). Relatedly, it is assumed by the practitioner that the client is the only one who can find the solutions to the problem, apply them and eventually solve them.

#### 3.3.2. Problems v. solutions

The second theme expands upon the central principle of shifting attention away from problems and onto solutions. The way problems and solutions are understood, defined and used within solution-focused approaches was a key principle. Within solution-focused approaches there was wide encouragement to minimize discussion of the problem and instead focus on solutions.

“Focus on solutions, strengths, and health. Solution-focused brief therapy focuses on what clients can do versus what clients cannot do. Instead of focusing and exploring clients’ problems and deficiencies, the focus is on the successes and accomplishments when clients are able to satisfactorily address their problems of living” (20, p. 3).

In this vein, the client and practitioner should focus on personal strengths to build upon, rather than spending time finding a way to overcome specific problems. It was perceived to be more useful to conceive, understand and elaborate on solutions than on problems (21). However, authors differed in the amount of emphasis that should be put on “problem-talk,” with some advocating that a problem should be clearly defined in order for solutions to be found, while others argued that problem-focused talk should be avoided altogether. However, it is acknowledged that it would be difficult to completely avoid problem-talk, and clients should be given space to discuss the problem that brought them to therapy if they wish to;

“In theory, the past (and the problem bringing the person to therapy) does not need to be addressed (however, it usually is, especially during the beginning of the first session during the transition from problem talk to solution-talk)” (22, p. 138).

Similarly, it was commonly suggested that a rapid or complete resolution of a client’s problems is unrealistic and instead the focus of therapy should be on the completion of small, obtainable goals in order to keep them moving forward. Some authors argued that it was still possible to reach a solution without fully understanding the problem, including how it is maintained or how it arose. And that solution-talk should far outweigh problem-talk.

“make a conscious effort to stay focused on solution-talk and deemphasize problem talk” (19, p. 37).

Solving problems is not the goal in this therapeutic approach and sometimes the solutions identified are not necessarily directly related to a particular problem.

“the development of a solution is not necessarily related to the problem; the client is the expert; if it is not broken, do not fix it; if something works, continue with it; if something does not work, do something else” (23, p. 88)

Overall, the client (and not the problem) should be at the center of the enquiry and this centralization should be maintained throughout the therapeutic process.

#### 3.3.3. Solution-focused tasks

There were clear tasks, techniques and processes that were detailed in the literature for practitioners to use in order to be solution-focused. These seemed to cluster around temporal dimensions related to the client, namely the past, present and future. These have been described in three separate sub-themes below.

##### 3.3.3.1. Past work

Past work primarily entailed identification of and reflection on existing strengths, and examples of exceptions to the identified problem. Exploring past successes and what the client has already done was seen to help clients identify their strengths and previous solutions that can be built upon or re-utilized. The identification of these existing strengths and resources related to the concept of client empowerment which was also present consistently in the included papers. Identifying existing client strengths contributes to a sense of resilience and hope, as solutions can be built using the strengths the client already possesses. This then empowers clients by reinforcing their sense of autonomy, increasing their confidence in dealing with the situation, and helping them to feel supported and optimistic about change and finding solutions.

The notion of searching for exceptions, i.e., identifying examples from the past of when the problem was not occurring, was an important component within the approach, being mentioned in most of the included papers.

“Search for ‘exceptions’; these are times when the problem could have occurred, but it did not or it was less severe than usual. The patient is encouraged to describe what was different when the problem did not occur, or what the client did differently that may, at least temporarily, have ameliorated the problem. The goal is for the client to repeat or do more of what has worked in the past and gain confidence in making improvements for the future” (24, p. 328).

Practitioners may ask clients questions such as “how did you do that?”, fostering a sense of the client’s involvement in this change of situation. This helps to change the client’s perspective of the situation as they are able to see that there are times when the problem is absent or less severe, and realize that situations are not fixed but are fluid. “Positive blame” a technique to attribute the exception to the role the client played in this rather than some external factor, was also referenced (17). Exceptions can be explored to identify what skills and resources the client used, which can then be used in the present to amplify what works for the client and contribute to the construction of a solution.

##### 3.3.3.2. Present work

Present work entails the practitioner using techniques to encourage the client to reflect and think about what can change in the present and to help them better understand their current situation. Perhaps one of the most integral aspects of this is the use of the scaling technique to quantify the client’s problems, with one article describing scaling questions as the “work horses” of solution-focused approaches due to the frequency in which the questions are asked and the ability to achieve a number of therapeutic ends (25). Scaling questions ask the client to rate a situation on a standardized scale, usually from 1 to 10, with 1 representing the worst that the situation could be and 10 representing the best (14). Scaling the clients desired outcome or how far they perceive themselves from their undesirable situation, provides a quantifiable measure of the current situation. It allows the practitioner and client to explore the client’s motivation to achieve their desired outcome as well as the level of control they possess over the current problems in their lives (26). This allows the client to take ownership over their current situation and identify what steps need to be taken to move forward to achieve their goal.

“Scaling - assigning a numeral value (typically between one and ten) to the individual’s perceived condition, improvement, mood, success, or other intangible (…) Scaling simplifies complex experiences by asking people to grade situations quantitatively” (27, p. 46).

Questions posed by the practitioner encourage the client to take the lead in applying the skills and resources identified in “past work” to their current situation. Successfully applying these strengths in the present encourages the client to see themselves as an individual capable of creating positive change in their life. These strengths and resources must be utilized and amplified throughout the present work, and successful behavior that has worked in the past repeated to encourage improvement of the present situation.

The identification of these strengths will contribute to the overall hope and possibility for positive change, as the client can continuously build upon their existing strengths to formulate solutions. However, it is imperative to continuously discuss whether the solution has created change in the client and not to simply focus on the solution being a good solution.

The principle of “if it’s not working, do something different” highlights the danger of clients becoming stuck in cycles of continued problem patterns while attempting solutions that do not manifest into change (28). Additionally, the same risk is true for the practitioner, who can get caught in similar “vicious circles” of continued rigid therapeutic techniques that attempt to guide their clients. The practitioner and client must free themselves from these cycles by challenging themselves to look for novel and personalized methods to support the achievement of the client’s goals, which are worked toward in “future work.”

##### 3.3.3.3. Future work

In addition to reflecting on the past and rating the present there was also a clear tradition within the selected research papers of orienting toward the future. This is used in a number of ways, but generally encapsulates thinking of a goal and then envisioning (and then generating) the solutions to achieve it. This makes up a large proportion of the time used within sessions, namely visualizing where the client wants to be and focusing on that preferred future.

Language plays an important role in envisioning this future and influencing the client’s perception of the goal. Language should be “*imaginative, positive, and creative*” (29, p. 18), and the narrative act of solution development should be semantically different to the language of describing a problem.

“The language of solution development is different from that needed to describe the problem” (28, p. 26).

As has been outlined earlier, questioning by the practitioner is key to helping the client work through these processes. The “miracle question” is a type of question that was referenced frequently and is described as a core technique used to help the client visualize and describe in some detail how they want things to be if the selected problem was absent. The “miracle question” can be framed in a variety of ways, but ultimately seeks to establish what a preferred future would be like for that individual. This is a way to immediately frame the therapy as solution-oriented and short-term.

“Inquire as to what will be happening in clients’ lives when the problem is no longer occurring. For example, [you] might ask, “Let’s say that while you are sleeping tonight a miracle occurs and your problem has disappeared. How would you notice that the problem was gone? What would you be doing differently? What would other people notice as different about you?” After a detailed description of change is developed, one that includes behavioral components (e.g., “How will you be acting differently?”), solution-focused [practitioners] and clients work together to “make the miracle happen” (30, p. 301).

Relatedly, change is also a key expectation of the approach, and links to how practitioners orient toward the client’s goals. Solution-focused approaches view change as both continuous and inevitable within the therapeutic process, but also in everyday life. There is an assumption that the client cannot help but change, and the client should be encouraged to articulate what they want to change and then focus on the steps needed to achieve this.

“An assumption of solution-focused therapy is that change is so much a part of living that clients cannot prevent themselves from changing” (31, p. 10).

Both the change-talk and the change processes are one of the most important goals within solution-focused approaches. But change needs to be iterative. Small, incremental changes inevitably lead to larger changes over time, so it is important that clients are not too ambitious in their aims. The focus should be on small, manageable and motivating steps and understanding what would be different if these more subtle changes occurred. Ultimately, these small steps will lead to more impactful change for the client.

“This principle leads us to seek small changes in therapy; changes which, albeit small, can lead to bigger changes in clients’ lives” (28, p. 26).

#### 3.3.4. Views of others

Another tool identified in our synthesis, used by solution-focused practitioners to encourage solution elaboration, was for the practitioner to use questioning to encourage the client to consider the views of others. The literature discusses several advantages to these conversations including the employment of a new lens with which to process situations, the development of new indicators of positive behavioral change, and enhanced future orientation.

“The person is asked to recall interactions with others and to assign significance to these interactions. The scales in this exercise are used to measure what others would observe about them from the past, the present, or future. The person, then, in theory, becomes more aware of the responses of others by envisioning their reactions, what others would want to see happen, and how the person could bring about the necessary change. Imagining how a third party would scale progress offers insights to the practitioner as well” (27, p. 46).

Reflecting on the views of others (most often significant others) during sessions was most often described as the use of “relationship questions”, one of the key types of questions in solution-focused approaches. Such relationship questions can be used following the aforementioned miracle question, thereby inviting the client to describe how others would recognize that they had improved or reached a higher level on the scale.

“It is also helpful to ask clients what their significant others think or might think about their problematic situation and progress (…) Examples are: "What would your mother (or husband, friend, sister, etc.) notice different about you if they didn't know that a miracle has occurred?" (32, p. 50).

Relationship questions encourage the client to assign significance to their interactions with others, and envision how to bring about the change necessary for others to notice they, or their situation, has improved (27). As the questions recognize the interactional aspect of client problems, the client is encouraged to offer contextually rich descriptions of how others would react to behavioral or situational improvements (20). The practitioner can also glean insights from the way in which the problem is defined and progress is scaled from a third-party perspective. Collaboratively, the practitioner and client are able to identify contextually relevant indicators of positive behavior that can be used to orient the client. These steps can then be used to move conversations from focusing on internal emotions to external manifestations of solution-focused behaviors and help clients move toward their preferred future.

#### 3.3.5. Practitioner characteristics

The final theme orients around descriptions within the included papers of particular traits and characteristics practitioners should demonstrate while working within a solution-focused approach. These were grouped into four sub-categories: “personal virtues”; “communication techniques”; “generic therapeutic principles”; and “principles which are related to solution-focused approaches.”

“Personal virtues” were traits commonly described as pertaining primarily to the practitioner’s individual approach, such as being friendly or respectful. These were traits that were likely to be needed by any practitioner no matter the therapeutic approach but were nonetheless referenced as important within solution-focused approaches.

“When clients were asked why they placed the therapist where they did, there were a variety of responses; ‘the therapist was fair-minded’, ‘the therapist was honest’, ‘the therapist was trustworthy’” (33, p. 41).

“Communication techniques” were also often listed as a key tool to ensure successful delivery of this approach. Although perhaps these could be seen as techniques used within all therapeutic approaches, such as active listening or selecting and tailoring language carefully, these techniques were also linked to the importance of ensuring a focus on solutions and strengths.

“A gentle, affirming, non-impositional but persistent listening style communicates empathic understanding, while also communicating a belief in the strengths of the client and in the possibility that they can make things different” (21, p. 386).

Language was seen as key in working collaboratively, orienting toward solutions, and maintaining the therapeutic principles key to a solution-focused approach.

Conversely, the generic therapeutic principles that were outlined, like objectivity and avoiding confrontation, were viewed as general good practice that is required from practitioners, regardless of the therapeutic approach adopted.

Finally, some of the characteristics that were commonly described were seen to be less generic and more related to the specifics of working within a solution-focused approach. These included assumptions the practitioner should hold about the client, such as viewing the client as the expert, and taking a not knowing stance. Quick (34, p. 532) describes the central tenet of the practitioner’s approach within a solution-focused model as:

“The model’s overarching philosophy of ‘doing what works and changing what doesn’t’, applies not just to the client but also to the therapist as he or she proceeds with the work. When a technique or emphasis on a particular component of the model appears to be helpful, the therapist may well choose to continue it; when a certain kind of inquiry does not appear to be helpful, the therapist should probably ‘do something different.”

This demonstrates the specific requirement of solution-focused practitioners to apply the principles to their own work and practice. This appears fairly unique to this approach, with the practitioner expected to be willing to abandon their own preferred techniques in favour of doing what works for the client. This demonstrates the collaborative nature of the approach - that its philosophy applies equally to both clients and practitioners and they construct the progression through therapy together.

### 3.4. What makes an approach solution-focused?

As part of our critical reflection on the synthesis process we developed Table 1, based on Waltz et al’s, Addis (35) model for assessing treatment distinctiveness. This helped us to organize our thoughts and understanding of what makes a solution-focused approach distinctive to other approaches, after having conducted the synthesis process. This was based on how the included papers’ authors described or presented the unique and distinctive components of the therapy, rather than direct comparisons with other therapeutic approaches.

**Table 1:** Treatment distinctiveness assessment for solution-focused approaches.

**Essential and unique**
(1) Identifying the client’s preferred future, usually using the miracle question
(2) The assumption that change is constant
(3) Identification of times when the problem is less severe (search for exceptions)
(4) Practitioner’s assumption that the client has all the resources and skills necessary to enact their preferred future
(5) The practitioner leads from “one step behind”
(6) Use of complimentary language by the practitioner
**Essential but not unique**
(1) Assigning homework
(2) Focus on identifying existing resources and strengths and amplifying them
(3) Questions exploring the views of others on the problem and the client’s progress
(4) Collaborative working between the practitioner and client
(5) Establishing a therapeutic alliance
(6) The client is seen as the expert on their experience
(7) Scaling of progress
**Acceptable but not necessary**
(1) Use of language-based tools during sessions by the practitioner (language matching, metaphors)
(2) Discussion of the problem’s causation
(3) Planning for termination
(4) Client leading the session
**Proscribed**
(1) Discussion of client deficits
(2) Clinician defines the problem and its causation

### 3.5. Conceptual framework

**Figure 4:**
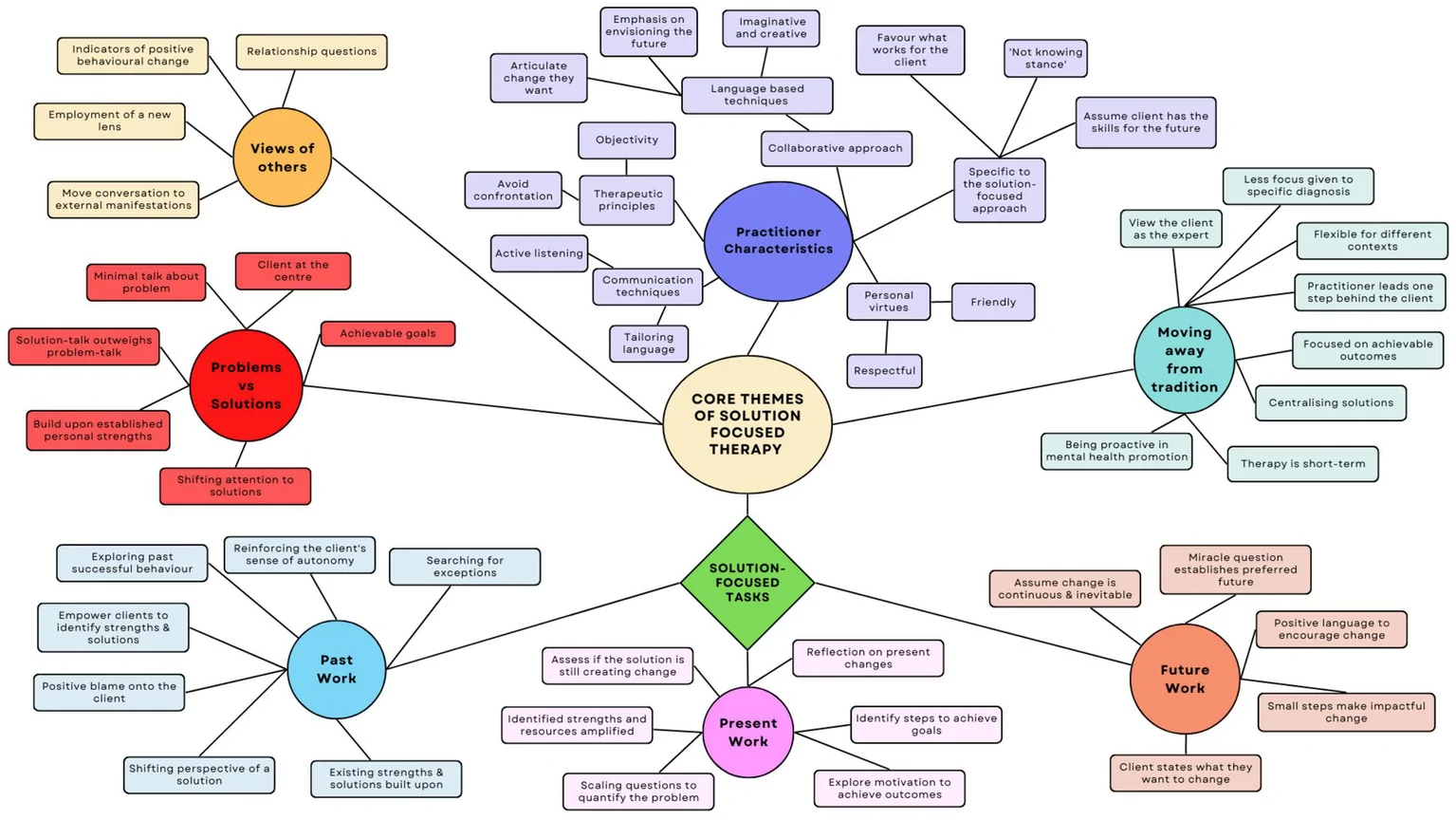
Conceptual framework resulting from our synthesis of the conceptualization of solution-focused approaches in the adult mental health literature.

Our synthesis and concept mapping resulted in the development of a conceptual framework (see Figure 4), showing our understanding of the conceptualization of solution-focused approaches in the adult mental health literature.

## 4. Discussion

Using a narrative approach, we have identified the common, and distinct, attributes of solution-focused approaches as they are described in the adult mental health literature. Despite solution-focused approaches encompassing numerous therapeutic models, not just SFBT, and being implemented in a wide variety of settings, their overall conceptualization appears relatively consistent over time even though some of its originally important techniques, such as compliments, are scarcely mentioned in more recent literature. While we identified several key components, none were described in every single paper included in this review. Table 1 details some of the common concepts and techniques described in the literature that we believe appear to characterize the definition of an approach as “solution-focused.”

Across the included papers, there were several themes that demonstrated stability in how solution-focused approaches are conceptualized: perceived as moving away from traditional approaches; problems versus solutions; solution-focused tasks; views of others; and practitioner characteristics. The notion of the client being an expert and becoming empowered through the therapeutic process appears consistently across the included papers and throughout the themes we developed. Similarly, the importance of collaborative working was emphasized, as was the assumption of change being inevitable and essential for clients to progress. Although there was some disagreement between the included papers on the extent to which problems should be discussed, there was agreement overall that the client rather than the problem is the center of enquiry. The principle of focusing on client’s solutions rather than their problems was more radical at the time of the approach’s conception, with this and other concepts now integrated into other therapeutic approaches. However, solution-focused approaches are still seen as more contemporary and distinct from more traditional psychotherapeutic approaches.

Despite our themes being stable in the literature over time, comparing the papers based on year of publication identified some differences in the specific components commonly described. In particular, more recent publications cited task-based components more frequently, such as the use of scaling questions and goal setting, perhaps reflecting the current trend of mental health services being more outcomes-based and focused on tangible measures of progress and recovery. This may be particularly relevant in the UK and other countries where health services are publicly funded. Moreover, the absence of references to “taking breaks,” a core component of the original formulation, in the literature may be a reflection of demands on resource-limited mental health services, where the number of service users is high and time is limited. Taking breaks may be seen as an inefficient use of this limited time, despite being integral to the original conceptualization of the approach. Overall, the grouping and comparison of papers by setting and publication year illustrated a subtle evolution in the way that solution-focused concepts are described in the literature, or at least which key components are given the most salience. The current structure and capacity of modern healthcare services may underpin this subtle change over time.

The results of the vote counting identified one of the main areas of disparity to be the theoretical background ascribed to the solution-focused approach, with most papers either citing de Shazer’s original approach or giving no theoretical background at all. de Shazer’s original description of solution-focused therapy was so influential that perhaps it is considered a theoretical background in and of itself, despite contemporaneous criticisms that he was atheoretical and too pragmatic (36). The persistent influence of, and reference to, de Shazer’s approach could explain why there is so much consistency in how later solution-focused approaches are conceptualized. That post-modern theories were the second most commonly referenced theoretical background is unsurprising given the conceptualization of solution-focused approaches as centring on the client being the expert, the important use of language, and their perceived move away from more traditional approaches. The numerous theories uniquely identified in individual papers could be a result of the flexibility and applicability of solution-focused approaches to a variety of different settings, allowing for theories relevant to those specific settings to also be ascribed to their particular approach.

Vote counting of the key definitions and steps within the papers identified many unique descriptions that were grouped into overarching categories to aid analysis. These categories demonstrated the myriad ways key concepts were applied. For example, most papers discussed the concept of “focusing on the client’s future,” but prescriptions on how to do this varied. While some advised the use of the miracle question and its variations (i.e., the tomorrow question), others suggested the adoption of a general outlook which is forward-looking and focuses on the client’s desired future. The most frequent steps and definitions were linked in some way to the notion of the client being at the center of enquiry. These included identifying client strengths, focusing on the client’s desired future, and collaborative working between the client and practitioner. The variability of techniques to implement solution-focused concepts may contribute to the enduring conceptualization of solution-focused approaches as being adaptable and appropriate across a wide range of settings and practitioner roles.

This variability in techniques also demonstrates the distinction between concepts we identified which appear core to the underlying mechanism of change within solution-focused approaches and are consistent over time and setting, such as a focus on the future, and the specific techniques used to enact these concepts, which varied. Our themes explored these concepts, whereas previous reviews of solution-focused approaches often simply describe the presence or absence of techniques. Therefore, our conceptual framework provides a basis for understanding how solution-focused approaches achieve their desired outcomes, and the purpose of the techniques commonly used within them. Despite the wide range of settings of our included papers, the concepts explored in our themes were consistently present. This suggests these concepts are key to formulating a solution-focused approach, regardless of the specific techniques then used. While we cannot claim to have assessed which concepts are necessary for a successful solution-focused approach, the enduring presence of such concepts suggest their essentiality to these approaches, and our framework could provide a basis for further investigation in future research.

### 4.1. Strengths and limitations

The review provides a succinct overview of the understanding and use of solution-focused approaches utilizing a rigorous and novel form of analysis. The use of several analytic techniques, recommended by Popay et al (9), allowed a holistic approach to analyzing the data. The vote counting proved a useful tool to establish an initial description of patterns arising from the included studies. This initial analysis then allowed for a more thorough and rich exploration and thematic analysis of the extracted data. At each stage of the analysis, several multi-disciplinary researchers were involved, allowing for different views to be incorporated into the final analysis. Another strength of this review was the inclusion of papers from the last 29 years. To include research that stems from the first published study on solution-focused approaches to the present allowed us to establish a historical as well as conceptual perspective of the approach.

However, the review is limited by the fact it only included studies published in the English language. To broaden the search to include publications in different languages could have enhanced or changed certain findings of the review. Second, the review was limited to include papers which specifically used the phrase “solution-focused,” as this was one of the search terms, and so may have missed papers which broadly used a solution-focused approach but was described using a different name. This may account for the consistency we found in our results. Moreover, this could be considered tautological since we searched for the phrase “solution-focused,” it is unsurprising we only found approaches self-described as “solution-focused.” However, as we were interested in looking at how the term “solution-focused” is used and understood in the mental health literature this was deemed appropriate for this review. Third, our synthesis of the findings was limited to the knowledge and understanding of the approach and other therapeutic approaches within the review team. However, discussion of our emerging findings on several occasions with a wider team of researchers and clinicians was beneficial for considering and incorporating other views and interpretations into our final synthesis.

### 4.2. Comparison with existing literature

The influence of the identified core principles of solution-focused approaches are visible in newly developed therapeutic practices. For example, variants of cognitive behavioral therapy (CBT) which are specifically designed to engender resilience and identify strengths within clients. These include strength based cognitive behavioral therapy (37) which draws on several elements which are essential to solution-focused approaches such as client strengths identification, tactful use of metaphors and language, and even the use of compliments and smiling by the practitioner to further encourage the client. Another example being DIALOG+, a therapeutic intervention incorporating steps based on the principles of solution-focused therapy, which is used globally (38). While the specific language of therapeutic tools such as the “miracle question” seem to remain unique to solution-focused approaches, there is a clear persevering influence of solution-focused components in later therapeutic developments.

There have been numerous critiques of solution-focused approaches in the four decades since their development. For example, Walker, Froerer and Gourlay-Fernandez (39) argue that the approach ignores the importance of discussions of emotional experiences, and in particular the ways in which emotional language can mobilize behavior change within the client by allowing them to identify emotional strengths. However, the potential to emphasize behavioral and cognitive changes, potentially to the detriment of emotional change, was not explored in the literature reviewed. Within descriptions of the tools which must be mobilized to improve therapeutic outcomes, emotional change is reduced to a product of the cognitive change inherent to solution-focused approaches as opposed to a catalyst in its own right. Furthermore, while the articles broadly discuss the importance of language and metaphors, the specific importance of emotional language during therapeutic sessions is scarcely mentioned and its role in solution-focused approaches requires further discussion.

Further, Stalker, Levene and Coady (40) note that because solution-focused approaches do not provide broad based contextual assessments, it may not be appropriate for severe conditions, where important contextual factors within the lives of the client may be overlooked, in part, due to the brief nature of the therapy. While some articles provided approximate timelines for courses of treatment, other articles noted that the conclusion of solution-focused sessions was a decision that depended on the opinions of the client regarding whether it was necessary. While this may be true for SFBT, the more general widespread adoption of solution-focused approaches in a variety of settings demonstrates its applicability for more severe conditions and its ability to be implemented in a variety of contexts and formats, such as its use in emergency care with individuals who self-harm (41). However, while generally personalization of treatment is emphasized across the literature, it does not explicitly address whether “doing what works” should encompass the use of more comprehensive specific initial assessments, or how a solution-focused approach can be maintained while such assessments are being completed with the client.

Although the literature reviewed celebrates the cultural sensitivity of solution-focused approaches as one of its key strengths, feminist criticisms of the approach argue that problems experienced by clients are rarely contextualized within cultural conditions for oppression. Such critiques note that treatment of the client as an expert may prevent cultural myths held by the client, e.g., about gender roles, from being challenged. While the literature positions solution-focused approach’s emphasis on personal responsibility and actions as being empowering to clients, Dermer, Hemesath and Russell (42) note this can be unhelpful to clients who are grappling with larger systems of influence on their lives which are intangible and not prone to change through individual actions. It is argued that instead of empowerment, discussions of personal responsibility can lead to clients feeling responsible for conditions which are inflicted upon them, thus making them feel deserving of oppression. In such cases, Dermer, Hemesath and Russell (42) state that explanation-oriented approaches are preferable to action-oriented approaches as they permit the use of “blame” and can mobilize gradual and sustained change toward improved futures. Throughout the literature reviewed, the theoretical underpinnings of solution-focused approaches often include the prioritization of cultural sensitivity without further scrutiny into how false cultural narratives are meaningfully challenged, without compromising the notion that the client is an expert on their own experiences.

### 4.3. Implications

The conceptual framework and synthesis resulting from this review provides a significant contribution to the literature by providing a comprehensive understanding and analysis of solution-focused approaches to adult mental health care. Given solution-focused approaches’ widespread use, these findings will be of interest to researchers and clinicians working with such approaches, to better understand the mechanisms by which their approach works, and how the key principles are utilized. The conceptual framework developed will be useful to refer to when describing these approaches in future research and in clinical practice.

Future research could seek to establish how emotional language is used within solution-focused approaches and the impact this has on therapeutic outcomes. Moreover, the investigation of solution-focused approaches in cultures other than western, English-speaking ones would first uncover whether there are any differences in how it is conceptualized, and second, whether the conceptual framework we identified is maintained while also addressing the needs of other cultural conditions within adult mental health care. It may also be beneficial for future conceptual work to investigate how the key concepts outlined in this paper present in other psychotherapeutic and multimodal approaches. This could identify areas of overlap between approaches, and whether the shift in practice to fit modern healthcare services is common across psychotherapeutic approaches.

### 4.4. Conclusion

The findings of this conceptual review demonstrate the relatively stable and coherent understanding and use of solution-focused approaches within adult mental health research. Despite being applied across a range of different clinical and geographical contexts, over the 40 years or so since the original formulation of the approach the way solution-focused approaches are conceptualized have remained similar. Perhaps this is unsurprising given that one of the core assumptions of the solution-focused approach is to ensure it is generic and holistic. The findings of this conceptual review will be helpful in providing practitioners and clinicians, particularly those who are new to the approach, a deeper understanding of the core principles and active mechanisms through which solution-focused interventions work.

## Author contributions

LJ ran the searches, removed duplicates, and collated the papers to be screened. LJ and JW performed both the title and abstract screening of texts, and full text screening. LJ, JW, PM, NA-H, and KE extracted data from the included papers and performed the analysis. LJ, PM, NA-H, and KE developed the conceptual map, the assessment of treatment distinctiveness, and wrote the final manuscript. All authors have approved the final manuscript.

## Funding

This work was supported by the National Institute for Health Research (NIHR) [TACK, RP-PG-0615-20010]. The views expressed are those of the author(s) and not necessarily those of the NIHR or the Department of Health and Social Care. The NIHR had no role in the study design, data collection, analysis, interpretation of data, writing of the report, or decision to submit for publication.

## Conflict of interest

The reviewer PP declared a past co-authorship with the author KE to the handling editor.

The authors declare that the research was conducted in the absence of any commercial or financial relationships that could be construed as a potential conflict of interest.

## Publisher’s note

All claims expressed in this article are solely those of the authors and do not necessarily represent those of their affiliated organizations, or those of the publisher, the editors and the reviewers. Any product that may be evaluated in this article, or claim that may be made by its manufacturer, is not guaranteed or endorsed by the publisher.

## Footnotes

^1^ https://www.crd.york.ac.uk/prospero/display_record.php?RecordID=90195
