# Supplementary material for: Solution-focused approaches in adult mental health research: A conceptual literature review and narrative synthesis
Source: Front Psychiatry. 2023 Mar 30;14:1068006. doi: 10.3389/fpsyt.2023.1068006 (PMC10098109; doi:10.3389/fpsyt.2023.1068006)
Supplement: Supplementary file 1 [file Table_1.DOCX]

Supplementary material 1. Details of all papers included in the review.

|  | **Author** | **Year** | **Country** | **Setting and Population** | **Interventional Investigative Research/Therapeutic Practice Research paper** | **Summary** |
| --- | --- | --- | --- | --- | --- | --- |
| 1 | M. Carrera, et al. | 2016 | Spain | Community mental health centre in Spain.  The population was clients who met the ICD-10 criteria for adjustment disorder and/or mild to moderate depression as the main clinical diagnosis. | Interventional Investigative Research Paper | The study aimed to assess the effectiveness of solution-focus group therapy (SFGT) through a pre/post study involving 132 mental health service users. Clients scored significantly lower scores on the depression and anxiety measures post-SFGT compared with pre-SFGT. |
| 2 | H. Ferraz and N. Wellman | 2008 | United Kingdom | Acute inpatient settings. Population of the review was on mental health nursing. | Interventional Investigative Research Paper | This review aimed to highlight the suitability and relevance for using solution-focused brief therapy (SFBT) in mental health nurse practice, with a focus given to acute inpatient settings. The papers included indicated positive changes in patient outcomes, therapeutic alliance, and nurse's goal-setting ability. |
| 3 | H. Ferraz and N. Wellman | 2009 | United Kingdom | Acute care setting; Population was drawn from the staff of three NHS psychiatric acute inpatient admission units and mental health day care setting in voluntary sector provider (London area). | Interventional Investigative Research Paper | This paper focused on testing staff on their knowledge, skill acquisition, and self-reported application of solution-focused brief therapy (SFBT) following a 2-day training. The staffs’ knowledge was measured prior to the training and then at 3- and 6-months post-training. The study concluded that the 2-day training was effective in increasing knowledge and understanding of SFBT in staff. |
| 4 | K. González Suitt, et al. | 2019 | Chile | Primary care in Santiago; Population was adult consumers of primary care services. | Interventional Investigative Research Paper | The study aimed to linguistically adapt SFBT to be used in a Latin American Country. Interviews were conducted to translate the main SFBT tools, as well as collecting information on the cultural needs that should be incorporated when delivering SFBT to individuals with alcohol use in primary care. |
| 5 | K. Isherwood and S. Regan | 2005 | UK | A residential rehabilitation service for people with severe and enduring mental illness; population was staff and service users drawn from the residential rehabilitation service. | Interventional Investigative Research Paper | The paper reported the use of solution-focused brief therapy (SFBT) approach in defining targets for improvement and change in service outcomes at a residential rehabilitation service. |
| 6 | J. S. Kim, et al. | 2018 | USA | Outpatient treatment for substance use disorders (Community addiction services); population was drawn from these services for individuals with a substance abuse disorder. | Interventional Investigative Research Paper | The study aimed to assess the effectiveness of solution-focused brief therapy (SFBT) in primary substance use treatment services for individuals with substance use disorders. A randomised control trial concluded SFBT group had similar results on the addiction severity index-self report and trauma symptom checklist as the control group who received research supported treatments. |
| 7 | A. Klingman | 2002 | Isreal | Junior High School Counselling Services; Population was the school counsellors. | Interventional Investigative Research Paper | The study focused on the use of solution-oriented approach to provide a solution for school counsellors to address their personal as well as professional concerns to decrease burnout and increase empowerment. The approach helped counsellors be more optimistic and self-confident about their professional role and within themselves. |
| 8 | J. Kramer, et al. | 2014 | Netherlands | PratenOnline chat; population was aged 12-22 years of age and had a CES-D score of 22 or higher. | Interventional Investigative Research Paper | The study aimed to evaluate the effectiveness of a web-based anonymous solution-focused brief therapy (SFBT) chat room for depression adolescents and young adults. The chat room significantly decreased depressive symptoms at 9 weeks and at 4.5 months compared with those individuals in the waiting list condition. |
| 9 | M. Mansouri, et al. | 2018 | Iran | Addiction Services (Opium Detox Centres); Psychology Centres; Population was drawn from the addiction services for individuals with an opium addition included. | Interventional Investigative Research Paper | The study focused on individuals with opium addition on their hopes and happiness. Eight solution focused therapy sessions were delivered, with the counselling improving the individuals hope and happiness. |
| 10 | M. McAllister, et al. | 2009 | Australia | In two major Departments of Emergency Medicine and acute care areas in South East Queensland; Population was the emergency nurses working in the study setting. | Interventional Investigative Research Paper | The study focused on the application of a solution-focused education intervention approach to provide clinicians with knowledge and skills to improve their response to patients who present because of self-injury. The intervention showed an expressed intention from the clinicians to act in a more person-centred and change oriented manner. |
| 11 | S. Northcott, et al. | 2015 | UK | At the University clinic; The population was individuals with any severity of expressive aphasia. | Interventional Investigative Research Paper | This paper focused on the impact solution-focused brief therapy (SFBT) can have on individuals with post-stroke aphasia on their psychosocial well-being. Individuals received between 3 and 5 therapy sessions, with the therapy showing encouraging trends in improving mood and communicative participation. |
| 12 | S. Priebe, et al. | 2019 | UK | Community mental health teams in the NHS; population was patients aged 18-65 years of age with a diagnosis of a SMI. | Interventional Investigative Research Paper | The paper aims to explore the use of DIALOG+, an intervention that uses a solution-focused approach, in three LMICs. Nine trials will be conducted over a 6-month period to assess the impact this approach has on individuals with severe mental health illnesses. |
| 13 | S. Priebe, et al. | 2015 | UK | Community mental health teams in the NHS; population was drawn from the staff and service users in NHS CMHT | Interventional Investigative Research Paper | The paper aimed to explore the effectiveness of DIALOG+, an intervention that uses a solution-focused approach, in community treatment for people with psychosis. Clinician and service-users were randomised to use DIALOG+ once per month over 6 months, with the results concluding DIALOG+ improved the quality of life in participants, as well as fewer psychopathological symptoms and better social outcomes. |
| 14 | S. Proudlock and N. Wellman | 2011 | UK | Clients for SFGT were under the care of a Crisis Resolution and Home Treatment Team (CRHTT). | Interventional Investigative Research Paper | The paper reported the use of solution-focused group therapy for adults with severe and enduring mental health disorders. Group therapy showed progress towards recovery as a significant increase in the Mental Health Recovery Measure was recorded post group therapy. |
| 15 | E. K. Quick and D. P. Gizzo | 2007 | USA | Permanente Psychiatry and Addiction Medicine service in San Diego; Population was drawn from individuals who will still receiving support from these services. | Interventional Investigative Research Paper | The study focused on the effect of solution-focused therapy groups on adult psychiatric outpatients. There was a strong correlation in the number of sessions attended with a greater sense of change. In addition, those who attended the group, perceived an increased ability to “control the problem”. |
| 16 | A. Seidel and D. Hedley | 2008 | Mexico | Participants were recruited from three institutions in Mexico: Alliant Interna tional University (Mexico campus); Jesus Maria (a Mexican NGO); and the Instituto Nacional de las Personas Adultas Mayores (a Mexican governmental institute for older adults); Population was drawn from individuals aged 60 years or older who were currently not in psychotherapy. | Interventional Investigative Research Paper | The paper reports of a preliminary study reporting the use of solution-focused brief therapy (SFBT) on older adults. Three sessions of SFBT focused on problems highlighted by the participants. Compared with the waiting list control group, the participants who had SFBT showed significant improvements. |
| 17 | J. Simon and T. Nelson | 2004 | USA | A community mental health clinic in Orange County, New York; population was drawn from adult clients of the clinic who have finished SFBT treatment. | Interventional Investigative Research Paper | The paper reports the responses of clients who reported their goal to have been reached using solution focused brief therapy (SFBT). Clients were asked what areas were helpful or any improvements required when delivering SFBT, as well as their therapeutic relationship. |
| 18 | M. Y. Lee, et al. | 2001 | USA | A large community mental health centre in a city in Midwest USA; population was drawn from therapists working at the centre. | Interventional Investigative Research Paper | The paper reports the findings of a pilot study that aimed to assess the effectiveness of solution-focused therapy (SFBT) for treating clients with depression. The clients received six sessions over a 6 month period. Findings indicated a significant decrease in clients’ depression as well as a significant improvement in the clients’ general functioning and satisfaction with their support network. |
| 19 | S. Wiseman | 2003 | UK | St Luke's Hospital, Middlesborough; population was patients who presented to the local general acute hospital with self-harm symptoms. | Interventional Investigative Research Paper | The paper describes the implementation of solution-focused brief therapy (SFBT) on how it altered the clincians response to patients with self-harm injuries, as well as the impact on the patients. Of the 40 patients who had SFBT, only one repeated deliberate self-harm within the six-month study period. |
| 20 | B. Ingersoll-Dayton et al | 1999 | USA | Two nursing home facilities, one not-for-profit, one for-profit; population was drawn from these facilities to include those aged 60 or over and had a medical diagnosis of irreversible dementia. | Interventional Investigative Research Paper | The study aims to assess the efficacy of a solution-focused approach for problematic behaviour of nursing home residents with dementia. Family members and nursing aides reported diminished problems in the residents behaviour. |
| 21 | A. Lohuis et al | 2017 | Netherlands | Tameij - a dutch healthcare organisation that supports intellectually disabled people in their life and work; population was drawn from staff members at the organisation. | Interventional Investigative Research Paper | The paper focuses on understanding how healthcare professionals comprehend learning and implementing solution-focused support. |
| 22 | J. M. Bakker, et al. | 2010 | The Netherlands | Psychiatrists delivering solution-focused brief therapy in practice. | Therapeutic Practice Research Paper | This paper provides an overview of how solution-focused brief therapy can be an effective and applicable addition to the current psychiatric practices, with specific focus on the psychiatrist’s viewpoint. |
| 23 | R. Banks | 2005 | United Kingdom | Solution-focused group therapy focusing on the group of clients and therapists | Therapeutic Practice Research Paper | The paper focuses on the use of solution-focused therapy in a group setting. It discusses the role of ‘conductor’ in keeping track of the sessions, as well as the composition of the group and the various steps the therapist needs to take when delivering solution-focused therapy. |
| 24 | F. P. Bannink | 2007 | The Netherlands | Outpatients receiving psychotherapy by psychotherapists | Therapeutic Practice Research Paper | This article provides an overview on the theoretical background to solution-focused brief therapy and explains the processes for change seen by clients. Additionally, the paper discusses solution focused based therapy as a more cost-effective alternative to problem-focused, including the training of therapists-in-training in SFBT methods to maximize success. |
| 25 | F. P. Bannink | 2008 | The Netherlands | Outpatients receiving psychotherapy; population drawn from the clients and therapists | Therapeutic Practice Research Paper | This article provides an introduction into the concept of post-traumatic success, with the use of anecdotes and exercises for both the clients and the therapists. The paper emphasises sources to improve resilience and for posttraumatic growth to occur, as well as highlight how the use of solution-focused perspective shifts the focus on hope and optimism to help their client go from posttraumatic stress to posttraumatic success. |
| 26 | L. Burton and J. Lent | 2016 | United States of America | School setting focusing on counsellors. | Therapeutic Practice Research Paper | This article makes the argument, through two case examples, that vision boards can be integrated into SFBT approaches during counselling and depicts the approach taken. |
| 27 | M. C. Chandler and W. H. Mason | 1995 | United States of America | Addiction Services; population drawn from addiction nurses. | Therapeutic Practice Research Paper | This paper focuses on traditional methods of addictions treatment as a way of confronting client’s denial are contrasted  with solution-focused therapy approaches. The need to highlight clients’ competencies to create necessary solutions is explained in detail in the context of nursing. |
| 28 | S. Chaudhry and C. Li | 2011 | United States | Identifying therapeutic interventions that suit cultural needs of Muslims/ Muslim Americans (USA); population drawn from individuals who are Muslim/Muslim American and families | Therapeutic Practice Research Paper | This article consolidates the available literature that discusses the appropriateness of solution-focused brief therapy to fit the cultural needs of Muslims/ Muslim Americans in the USA. The literature provides support that SFBT is culturally sensitive for this target population and its shows potential efficacy for individuals who are Muslim. |
| 29 | T. Davidson | 2014 | USA | Use of solution-focused brief therapy during counselling sessions; population drawn from counsellors. | Therapeutic Practice Research Paper | The paper explains the STRENGTH acronym and the systematic approach it denotes for the integration of solution oriented and strength-based counselling principles. As both theory and practical checklists are incorporated into the acronym, the author deconstructs this acronym, referring to solution-oriented thinking and practical tools throughout. |
| 30 | S. de Castro and J. T. Guterman | 2008 | USA | Focus is on marital and family therapy; Population drawn from families who are coping with suicide. | Therapeutic Practice Research Paper | First, the nature and incidence of suicide is depicted along with consideration of the effects suicide has on families and prevailing treatment approaches for families coping with suicide. Next, the theory and practice of solution-focused therapy is described. Then, three case examples illustrate the application are presented. Finally, implications are discussed pertaining to the theory, practice, and research of solution-focused therapy for families coping with suicide. |
| 31 | S. de Shazer and L. Isebaert | 2004 | Belgium | Inpatient and outpatient settings; population drawn from individuals receiving alcohol treatment. | Therapeutic Practice Research Paper | This article provides an overview of a new approach to the treatment of problematic drinking developed at St. John’s Hospital, Bruges, Belgium. The program is based on Solution-Focused Brief Therapy and offered in both an inpatient and outpatient setting. Four-year follow-up telephone interviews were conducted for 118 inpatients and 72 outpatients who had completed the program; 84% of the inpatients and 81% of the outpatients reported maintaining their goals of either abstinence or controlled-drinking four years after completion of the program. The effectiveness of using a Solution-Focused Brief Therapy model for treatment of alcohol abuse is explored. |
| 32 | P. Dejong and S. D. Miller | 1995 | USA | Setting is within social work; population drawn from clients receiving therapy from their social worker. | Therapeutic Practice Research Paper | The paper details interviewing questions that a social worker can implement to discover their clients’ strengths in relation to their set goals. These questions are based upon the solution-focused approach to interviewing, that includes using the “miracle question”, scaling and exception-finding questions and coping questions. The relationship between those questions mentioned and the underlying concepts of identifying strengths within the client is explored. |
| 33 | J. S. Fleming and B. Rickord | 1997 | USA | Setting is within managed care; Population drawn from therapists working in those mental health care facilities | Therapeutic Practice Research Paper | This article provides an exploration into the historical events that showcased the need to use treatment methods capable of developing low-cost, quality mental-health services. An overview of the current literature is provided to support this need. The Solution Focused Brief Model is presented as one possibility of service delivery in mental health care. This model has been designed to meet the mental health needs of individuals, couples, and families for which effective brief therapeutic intervention is required. The paper also provides a basic overview of the theoretical underpinnings of the model, defined and synthesized into an applied approach. Additionally, examples of each aspects of the model is depicted for immediate use with clients. |
| 34 | G. J. Greene, et al. | 2006 | USA | Setting is in social services; population drawn from consumers who have a severe mental health disability | Therapeutic Practice Research Paper | This article delves into how the perspective of and techniques from solution-focused therapy can be used to further operationalize the strengths perspective for these direct, one-on-one interactions to facilitate mental health consumer recovery, as it has been reported that a strengths-based approach to case management has been concluded to be supportive of the mental health consumers experiencing recovery. |
| 35 | G. J. Greene, et al. | 1998 | USA | Focus on social work in a therapeutic context | Therapeutic Practice Research Paper | This article discusses a modification of the miracle question called the dream question. As a result of the dream question, clients can discover the solutions they are seeking within themselves and, thus, have a greater sense of personal power and Internal locus of control. Experience with the dream question thus far was deemed quite positive. |
| 36 | G. J. Greene, et al. | 1996 | USA | Setting is the crisis intervention system; population drawn from individuals in crisis. | Therapeutic Practice Research Paper | This paper discusses the applicability of solution-focused therapy, in terms of the strengths perspective, for use in crisis intervention. The “strengths” perspective differs to the current standard crisis intervention as it deems the clients already having the needed resources and coping mechanism to manage their problems; however, it is viewed that the client is not using them, not using them fully or they are not aware of using their resources. |
| 37 | J. T. Guterman | 1996 | USA | Mental health counsellors working with clients | Therapeutic Practice Research Paper | The paper firstly provides detail on the social constructionist, epistemological underpinnings of SFC are reviewed. Second, the underpinning clinical theory of SFC is detailed as well as the. clinical process of the model is explained, with case examples discussed. |
| 38 | B. F. Hagen and D. L. Mitchell | 2001 | Canada | Inpatient settings; population drawn from thought disordered clients | Therapeutic Practice Research Paper | The article presents an overview of solution-focused therapy, with a focus on how solution-focused techniques can potentially be applied to an inpatient psychiatry setting with clients with serious mental illnesses. Three case studies are included within the paper that demonstrates the application of SFT with clients experiencing thought disorders and provide a conclusion of the effectiveness of the SFT techniques. |
| 39 | C. Iveson | 2002 | UK | Therapists working with clients | Therapeutic Practice Research Paper | The article provides an overview of solution-focused brief therapy, exploring the use of this therapy for use in modern psychiatry. |
| 40 | J. S. Kim and C. Franklin | 2015 | USA | Setting whereby therapists use solution-focused brief therapy on clients. | Therapeutic Practice Research Paper | This paper explores how solution-focused brief therapy has conceptualised emotional processes and how therapists must use positive emotions to build solutions with their clients. It further explores Fredrickson’s broaden-and-build theory of positive emotions and provides empirical evidence for the importance of positive evidence in the change processes of psychotherapy and within SFBT. |
| 41 | D. J. Kiser, et al. | 1993 | USA | Setting whereby therapists use solution-focused brief therapy on clients. | Therapeutic Practice Research Paper | The author provides an overview the role of emotions has within solution-focused therapy and details multiple solution-focused interventions that use client affect. |
| 42 | C. Knight | 2004 | USA | Setting whereby therapists use solution-focused brief therapy on clients. | Therapeutic Practice Research Paper | In this article, the basic principles and techniques of solution-focused therapy are identified. Consistent with the notion of “parallel process,” the relevance and application of solution-focused strategies for supervision are examined. Case examples illustrate how solution-focused techniques can be successfully incorporated into clinical practice and supervision.  The paper depicts the basic principles and techniques of solution-focused therapy, as well as the applicability and incorporation of solution-focused strategies in clinical practice through case examples |
| 43 | D. C. Kondrat and B. Teater | 2012 | USA | Setting is the emergency department; population drawn from suicidal patients. | Therapeutic Practice Research Paper | This article details how the use of solution-focused therapy provides one avenue for assessing suicide risk and how the therapeutic intervention, which has not been subjected to the scrutiny of empirical research, can serve as an opportunity for increasing hope.  This paper articulates how solution-focused therapy can provide one method for assessing suicide risk and how this form of therapeutic intervention can be utilised as an avenues for increasing hope within these patients. |
| 44 | M. Y. Lee | 2013 | USA | Setting is within social work; population drawn from social workers | Therapeutic Practice Research Paper | The article provides an overview of the use of solution-focused brief therapy as a treatment model used in social work practice. SFBT holds the client accountable rather than responsible for their problems, while deliberately using language and symbols of “solution and strengths” to create positive and long-lasting change. |
| 45 | J. M. Linton | 2005 | USA | Mental health and substance abuse clinics; population drawn from mental health counsellors and those with substance abuse | Therapeutic Practice Research Paper | This paper explores the use of solution-focused counselling as an effective treatment option for those with addiction or substance abuse. A brief overview of traditional substance abuse treatment is offered, then the key assumptions of solution-focused counselling are then provided, and differences from traditional approaches are delineated. Benefits of using solution-focused counselling with substance abuse issues, such as the collaborative and client-centered nature of the approach, are presented; and sample interventions are described. |
| 46 | H. Matto, et al. | 2003 | USA | Mental health and substance abuse clinics; population drawn from mental health counsellors | Therapeutic Practice Research Paper | The paper discusses the compatibility of art therapy and solution-focused treatment and how these two methods can be integrated for the treatment of substance abuse. Ideas for solution-focused art therapy directives have been provided, as has a verbal processing protocol. This protocol identifies the critical areas to explore in relation to the client’s artwork. |
| 47 | M. McAllister | 2010 | Australia | Focus on mental health nursing with population drawn from mental health nurses. | Therapeutic Practice Research Paper | This paper expands on the Solution Focused Nursing model, showing how it connects to public health principles and develops the mental health nurse’s role – particularly in those clinical areas that require more than medical management and illness stabilization. |
| 48 | E. E. McCollum, et al. | 2003 | USA | Mental health and substance abuse clinics; population drawn from mental health counsellors | Therapeutic Practice Research Paper | This paper reviews the literature on Motivational Interviewing and Solution- Focused Therapy, argues for the usefulness of SFBT in group format, briefly describes a format for SFGT, and provides a case example of a SFGT session. |
| 49 | G. Miller | 1997 | USA | Focus on the use of solution-focused therapy with families. | Therapeutic Practice Research Paper | The author describes how brief therapy has evolved in the past 10 to 15 years from ecosystemic to solution-focused brief therapy. SFBT is characterized as a radically constructivist approach to personal problems which emphasizes how troubles and solutions are socially constructed realities. |
| 50 | E. Quick | 1998 | USA | Counselling; population drawn from therapists working with clients | Therapeutic Practice Research Paper | This paper explores the techniques and principles of the solution-focused therapy, with this approach emphasising ‘to continue to repeat positive behaviours and change those that don’t’ |
| 51 | M. D. Reiter | 2007 | USA | Counselling; population drawn from therapists working with clients | Therapeutic Practice Research Paper | This article explored the effectiveness of tasks set by solution-focused therapists, the expectations of conducting the therapy and how it can lead to change within the clients. Then the paper outlines the history of Solution-Focused Formula Tasks as well as the primary principles that will make change occur within the clients. |
| 52 | S. A. Schott and L. M. Conyers | 2003 | USA | Psychiatric Rehabilitation Units; population drawn from individuals with severe mental illness. | Therapeutic Practice Research Paper | The article refers to the implementation and effectiveness of solution-focused therapy strategies in a psychiatric rehabilitation setting. The literature concludes that solution-focused therapy complements the therapies of psychiatric rehabilitation units and that it is a potential intervention that can be used in recovery for empowering individuals with severe mental illness. |
| 53 | J. Sharry, et al. | 2002 | Ireland | Counselling; population drawn from therapists working with clients who are suicidal/self-harming | Therapeutic Practice Research Paper | The article outlines the principles of solution-focused therapy and the applicability to clients who are suicidal/self-harming. It states how solution-focused perspective can be used to assess and manage suicide risk, particularly using the scaling question. |
| 54 | T. Wand | 2010 | Australia | Mental Health Nursing; mental health nursing with clients | Therapeutic Practice Research Paper | The article outlines the key principles of solution-focused therapy and the commonalities with current core values with nursing practices. Clinical examples were provided to depict the application of solution-focused therapy in mental health nurse practice. |
| 55 | L. Zatloukal, et al. | 2019 | Czech Republic | Counselling; population drawn from therapists working with clients | Therapeutic Practice Research Paper | This paper explores two situations of working with metaphors in solution-focused brief therapy that can be used with clients: first the metaphors being offered by the client and second is working with metaphors offered by the therapist. Guidelines and practical examples are provided throughout the paper. |
| 56 | M. D. Reiter | 2010 | USA | Counselling; population drawn from therapists working with clients | Therapeutic Practice Research Paper | The paper depicts how hope and expectancy plays in the role of solution-focused brief therapy, with specific focus on the current model of psychotherapy by Trepper et al (2006) and the effectiveness in practice. |
