# Supplementary material for: Solution-focused approaches in adult mental health research: A conceptual literature review and narrative synthesis
Source: Front Psychiatry. 2023 Mar 30;14:1068006. doi: 10.3389/fpsyt.2023.1068006 (PMC10098109; doi:10.3389/fpsyt.2023.1068006)
Supplement: Supplementary file 2 [file Table_2.DOCX]

Supplementary material 2. List of theoretical backgrounds described in the included papers, identified through vote counting.

| Theory named | No. of mentions | Theory named | No. of mentions |
| --- | --- | --- | --- |
| Solution-focused | 18 | Post-modern | 13 |
| Ericksonian hypnotherapy | 4 | Strengths perspective | 4 |
| Hope theory | 2 | Systems perspective | 2 |
| Humanism | 1 | Behaviourism | 1 |
| Possibility theory | 1 | Ockham’s Razor | 1 |
| Empowerment based | 1 | Locus of control | 1 |
| Broaden and build | 1 | Holism | 1 |
| Strategic approach | 1 | Trauma counselling | 1 |
| Motivational interviewing | 1 | Late Wittgensteinian philosophy | 1 |
| Narrative therapy | 1 | Bioinformation theory | 1 |
